# Immunocastration and testosterone supplementation alter testicular development and muscle collagen gene expression in male chickens

**DOI:** 10.1016/j.psj.2026.107932

**Published:** 2026-10-09

**Authors:** Xiao Zhang, Kota Hoshina, Miyu Uemori, Shiro Egawa, Shotaro Nishimura, Yoshinao Z. Hosaka

**Affiliations:** a Laboratory of Functional Anatomy, Department of Animal and Marine Biosciences, Graduate School of Bioresource and Bioenvironmental Sciences, Kyushu University, Fukuoka, 819-0395, Japan; b Laboratory of Functional Anatomy, Department of Bioresource Sciences, Faculty of Agriculture, Kyushu University, Fukuoka, 819-0395, Japan

**Keywords:** Collagen, Immunocastration, Layer-type chicken, Meat quality, Testosterone

## Abstract

Immunocastration using a gonadotropin-releasing hormone (GnRH) vaccine offers a potential alternative to surgical castration in poultry, yet its effects on growth performance and meat quality in chickens remain poorly characterized. Thirty-six male Sakura chickens were allocated to three groups (n = 12): control (C), immunocastration with Improvac at 4 and 8 wk (I), and immunocastration plus subcutaneous testosterone implantation at 8 wk (T). Birds were sampled at 10 and 16 wk. Immunocastration induced anti-GnRH antibody production in both I and T groups compared with C (P < 0.001 and P < 0.01, respectively). At 16 wk, serum testosterone was lower in I than in C (P < 0.05), whereas T showed levels comparable to C. Gonadosomatic index was markedly reduced in T at 16 wk compared with both C and I (P < 0.001 and P < 0.01, respectively), possibly reflecting combined HPG axis suppression by immunization and negative feedback by exogenous testosterone. Body weight was higher in C than in I and T from 7 wk onward (P < 0.01), but overall average daily gain did not differ among groups. Muscle color (L*, a*) differed between groups in the thigh but not in the breast. Raw shear force of the iliotibialis lateralis (ITL) was lower in I than in C and T (P < 0.05 and P < 0.001, respectively), suggesting that immunocastration may reduce connective tissue stiffness in thigh muscle. In the breast muscle, *COL3A1* mRNA expression was elevated in T compared with C and I (P < 0.001 and P < 0.01, respectively), suggesting that testosterone may influence collagen gene expression in a muscle-specific manner. Further optimization of vaccination protocols specific to chickens is warranted to improve the consistency and efficacy of immunocastration effects.

## Introduction

Castration is widely used in livestock production to suppress aggressive behavior, improve meat quality, and facilitate management of male animals (Thompson, 2000). In male chickens, surgical caponization has long been practiced to modify carcass composition and meat quality (Symeon et al., 2012), but the procedure raises animal welfare concerns, prompting interest in immunocastration as a non-invasive alternative (Thompson, 2000; Quaresma et al., 2017). In layer-type chickens, the fate of male chicks has also become a subject of international concern; because they cannot lay eggs and show poor growth rates and feed conversion compared with broiler strains, they are typically culled at hatch (Murawska and Bochno, 2007; Krautwald-Junghanns et al., 2018). Rearing layer-type males for meat therefore remains economically disadvantageous under current production systems, and improving meat quality traits alone is unlikely to overcome this constraint. Nevertheless, basic information on how the reproductive physiology and meat characteristics of layer-type males can be modified is needed to evaluate possible alternative uses of these birds, such as capon-type production (Symeon et al., 2012).

Immunocastration exploits active immunization against gonadotropin-releasing hormone (GnRH), the master regulator of the hypothalamic-pituitary-gonadal (HPG) axis. Anti-GnRH antibodies neutralize endogenous GnRH and suppress pituitary secretion of luteinizing hormone (LH) and follicle-stimulating hormone (FSH), leading to testicular atrophy and a marked decline in circulating testosterone; unlike surgical castration, the effect is reversible (Thompson, 2000).

In swine, the GnRH vaccine Improvac is used commercially to eliminate boar taint by suppressing testicular steroidogenesis, and also improves growth performance (Dunshea et al., 2001).

In chickens, however, evidence for immunocastration remains limited. Wang et al. (2019) showed that Improvac suppressed spermatogenesis and serum testosterone in male broilers. Quaresma et al. (2017) evaluated immunocastration as an alternative to caponization and reported effects on body and bone development as well as meat color and composition. However, the effects of immunocastration on connective tissue metabolism and collagen gene expression in chicken muscle have not been examined. Furthermore, vaccination protocols optimized for swine may not translate directly to chickens.

Testosterone exerts anabolic effects on skeletal muscle (Herbst and Bhasin, 2004) and may influence collagen metabolism. Incorporating an immunocastration plus testosterone supplementation group therefore provides a means to dissociate effects attributable to testosterone deficiency from those arising from broader HPG axis suppression, including loss of LH and FSH signaling. The present study investigated the effects of immunocastration and testosterone supplementation on anti-GnRH antibody production, testicular development, serum testosterone concentrations, growth performance, meat quality, and collagen-related gene expression (*COL1A1* and *COL3A1*) in male Sakura chickens (a Japanese commercial layer strain), with the aim of providing basic biological data on the responses of layer-type male chickens to immunocastration.

## Materials and methods

### Animals and experimental design

All experimental procedures were approved by the Animal Experiment Ethics Committee of Kyushu University (approval numbers: A24-348 and A25-376). A total of 36 male Sakura chickens (a Japanese commercial layer strain) were obtained at 1 d of age from a commercial hatchery (Goto Hatchery, Fukuoka, Japan) and housed at the Poultry Experimental Facility of the Faculty of Agriculture, Kyushu University. After brooding, birds were reared under controlled conditions (27°C, 65% relative humidity, 12 h light:12 h dark cycle) with ad libitum access to feed and water. A commercial diet (Power Chick; JA Zen-Noh, Tokyo, Japan; starter to 4 wk, grower to 8 wk, finisher thereafter) was provided throughout the experiment.

At 4 wk of age, birds were allocated to three groups (n = 12 per group) based on equalized body weight: a control group (C) receiving subcutaneous saline injection; an immunocastration group (I) receiving subcutaneous injection of a GnRH vaccine (Improvac; Zoetis Japan, Tokyo, Japan); and an immunocastration plus testosterone supplementation group (T) receiving Improvac injection with subcutaneous testosterone implantation at 8 wk. A booster injection of Improvac or saline was administered at 8 wk of age in all groups. At each vaccination, Improvac (containing 0.4 mg of 2–10-GnRH analogue–diphtheria toxoid conjugate per 2 mL, i.e., 200 µg/mL, according to the package insert of the product marketed in Japan) was injected subcutaneously into the dorsal neck at 0.6 mL/kg body weight (120 µg conjugate/kg), and control birds received the same volume of saline; the booster was injected at a site different from that of the primary injection. The dose was determined with reference to previous studies in broilers that used fixed doses of 0.2 or 0.6 mL per bird (Quaresma et al., 2017; Wang et al., 2019) and to our preliminary experiments. In these experiments, a fixed dose of 0.6 mL per bird did not sufficiently suppress testicular development in Sakura chickens; when doses of 0.3, 0.6, and 1.2 mL/kg body weight were compared, GSI was reduced at 0.6 mL/kg, whereas body weight gain was suppressed at 1.2 mL/kg (unpublished data). Because no immunocastration protocol has been established for chickens, the injection interval followed the manufacturer’s recommendation for swine (two doses at least 4 wk apart), and the primary injection was given before sexual maturation. Testosterone implants were prepared by packing 0.07 g of testosterone (Fujifilm Wako Pure Chemical, Osaka, Japan) into silicone tubing (inner diameter, 2.0 mm; outer diameter, 3.0 mm; 40 mm length; Kaneka, Osaka, Japan) sealed with adhesive and inserted subcutaneously in the dorsal cervical region at 8 wk.

Birds were sampled at 10 wk (n = 5 per group) and 16 wk (n = 7 per group) of age under isoflurane anesthesia followed by cervical blood collection and euthanasia. The 10-wk sampling was intended to assess the gonadal response during the early phase of testicular growth, and more birds were allocated to 16 wk, the main endpoint for meat quality analyses. No a priori power analysis was performed; the total number of birds was determined by the capacity of the experimental facility.

### Measurements

Body weight was recorded weekly from 0 to 16 wk. Feed intake was measured daily per pen (one pen per group; 12 birds per pen until 10 wk and 7 birds per pen thereafter), and average daily gain (ADG) and feed conversion ratio (FCR) were calculated for the entire experimental period. At each sampling, testes were excised and weighed; gonadosomatic index (GSI) was calculated as bilateral testis weight / body weight × 100. Breast muscle (pectoralis) and thigh muscle (iliotibialis lateralis; ITL) were collected and used for subsequent analyses.

Serum testosterone concentrations were measured by enzyme-linked immunosorbent assay (ELISA; ADI-900-065, Enzo Life Sciences, New York, USA) according to the manufacturer's protocol. Serum was diluted 20-fold prior to analysis. Anti-GnRH antibody concentrations were quantified using a chicken anti-GnRH ELISA kit (MBS9912676, MyBioSource, California, USA) without dilution. Because of budgetary constraints, both assays, as well as the gene expression analysis described below, were performed on samples collected at 16 wk from the 4 birds per group whose GSI values were closest to the group median. Serum testosterone was also measured at 10 wk (n = 5 per group).

Muscle color (L*, a*, b*) was measured using a spectrophotometer (Color Spec, Zhejiang, China) at 3 sites each on fresh cut surfaces of the breast muscle and the ITL, and on the lateral and medial surfaces of the thigh musculature after skin removal. Moisture content was determined as weight loss after freeze-drying for 72 h. Shear force was measured on raw and cooked (75°C, 30 min water bath) muscle samples (2 × 2 × 1 cm) using a texture analyzer (Yamaden, Tokyo, Japan).

Total RNA was extracted from breast and ITL muscle using ISOGEN II (Nippon Gene, Tokyo, Japan) with a bead homogenizer, followed by isopropanol precipitation. RNA concentration and purity were assessed spectrophotometrically (Thermo Fisher Scientific, Tokyo, Japan) and adjusted to 1,000 ng/µL. Complementary DNA was synthesized using a reverse transcription kit with gDNA removal (Toyobo, Osaka, Japan). Real-time PCR was performed using TB Green reagent (Takara Bio, Shiga, Japan) with the following thermal profile: 95°C for 30 s, followed by 40 cycles of 95°C for 15 s and 58°C for 30 s. Melt curve analysis confirmed single amplification products for all primer pairs. The following primers were used: *GAPDH* (reference gene; NM_204305), forward 5′-GAGGGTAGTGAAGGCTGCTG-3′, reverse 5′-CATCAAAGGTGGAGGAATGG-3′ (113 bp); *COL1A1* (NM_204428), forward 5′-GAGCGACGGCTTCCAGTTTGAG-3′, reverse 5′-GTGTCGTGGTCCATGTAGGC-3′ (153 bp); *COL3A1* (XM_046921185.1), forward 5′-GCCAGTGGGAACGTTAAAA-3′, reverse 5′-ACCCCATTCACCAGTGTGTT-3′ (132 bp). Gene expression was normalized to *GAPDH*.

### Statistical analysis

Data were analyzed by one-way or two-way ANOVA followed by Tukey's multiple comparison test using Prism software (GraphPad, Massachusetts, USA). Differences were considered significant at P < 0.05. Data are presented as mean ± SEM.

## Results and discussion

Immunocastration with Improvac induced an anti-GnRH antibody response in both I and T groups compared with C (P < 0.001 and P < 0.01, respectively; Fig. 1F). To our knowledge, anti-GnRH antibodies have not previously been measured in Improvac-treated chickens, in which only downstream endocrine and reproductive effects have been reported (Quaresma et al., 2017; Wang et al., 2019). At 16 wk, serum testosterone was lower in I than in C (P < 0.05; Fig. 1D), whereas the T group showed testosterone concentrations comparable to C. Serum testosterone in T was numerically, but not significantly, higher than in I. Because the present design included neither a testosterone-only group, a baseline sample before supplementation (8 wk), nor more frequent sampling, and because only 4 birds per group were analyzed, these data indicate a numerical recovery of peripheral testosterone rather than a demonstrated restoration. At 10 wk, serum testosterone did not differ among groups (P > 0.05; Fig. 1B). This may reflect the time required for antibody titers to reach suppressive levels (anti-GnRH antibody was not measured at 10 wk) or the generally low testosterone concentrations before sexual maturation, as suggested by the lower values in C at 10 wk than at 16 wk. At both 10 and 16 wk, serum testosterone in T was not higher than in C, suggesting that the implant did not produce supraphysiological concentrations at these time points.

**Fig. 1:**
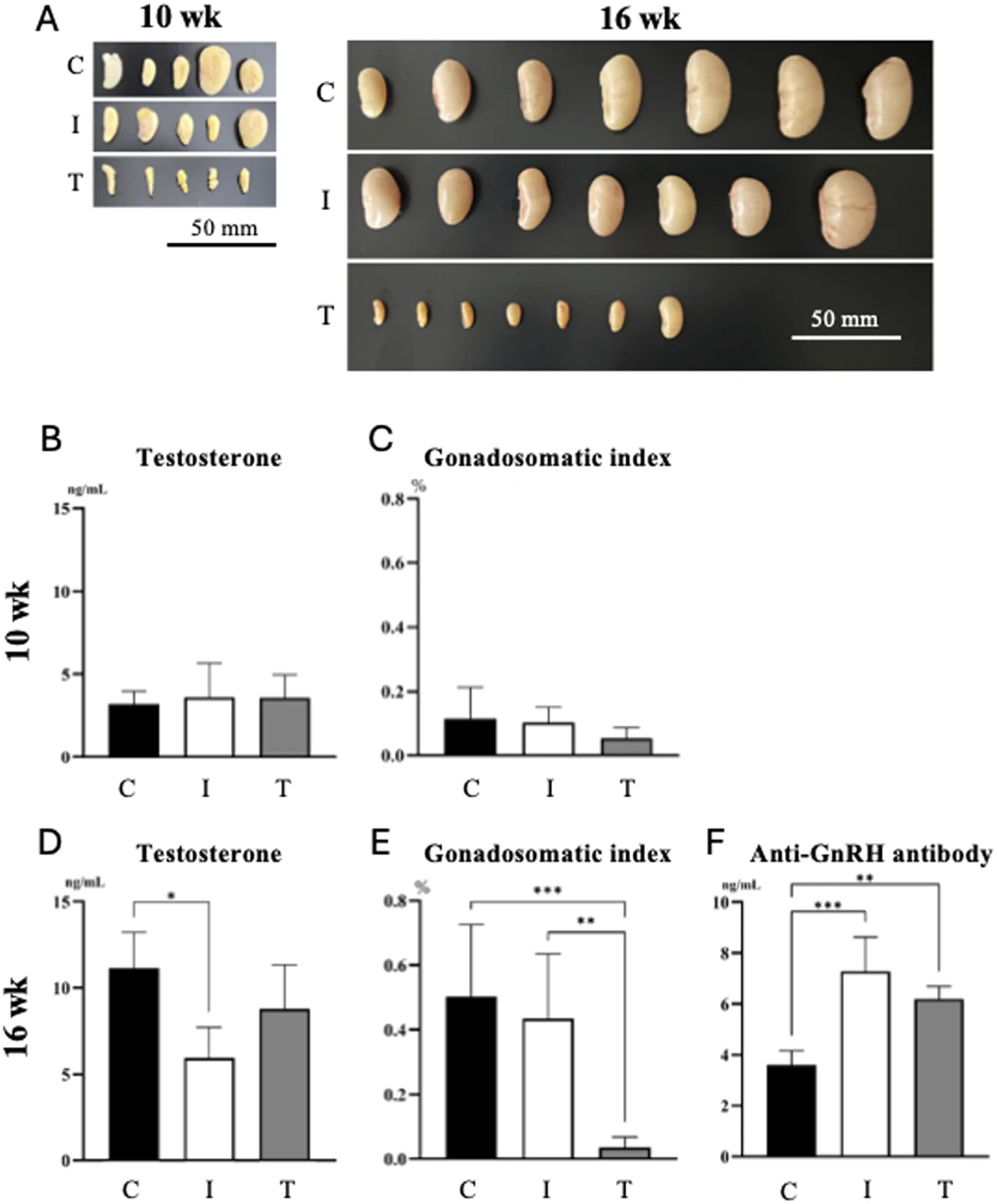
Reproductive organ development and endocrine profile in male Sakura chickens following immunocastration and testosterone supplementation. (A) Photographs of the right testes of all birds sampled at 10 wk (n = 5 per group) and 16 wk (n = 7 per group). Scale bar = 50 mm. (B, C) Serum testosterone concentrations (ng/mL; B) and gonadosomatic index (GSI; bilateral testis weight/body weight × 100; C) at 10 wk (n = 5 per group). (D–F) Serum testosterone concentrations (ng/mL; D; n = 4 per group), GSI (E; n = 7 per group), and serum anti-GnRH antibody concentrations (ng/mL; F; n = 4 per group) at 16 wk. Anti-GnRH antibody concentrations were measured only at 16 wk. Data are presented as mean ± SEM. Asterisks indicate significant differences between groups (Tukey’s test, *P < 0.05; **P < 0.01; ***P < 0.001). C, control; I, immunocastration; T, immunocastration plus testosterone supplementation.

At 10 wk, testes in the T group appeared macroscopically smaller than those in the C and I groups, although GSI did not differ significantly among groups (P > 0.05; Fig. 1C). By 16 wk, testes in C had enlarged substantially, those in I showed variable development, and those in T remained consistently small. GSI at 16 wk was markedly reduced in T compared with both C and I (P < 0.001 and P < 0.01, respectively; Fig. 1E), despite serum testosterone concentrations comparable to C in this group. This apparent paradox may reflect negative feedback of exogenous testosterone on residual gonadotropin secretion, compounding HPG axis suppression (Thompson, 2000). However, because exogenous androgen alone can suppress gonadotropin secretion and smaller testes in T were already macroscopically evident at 10 wk, 2 wk after implantation (Fig. 1A), the present design without a testosterone-only group cannot distinguish the contribution of immunization from that of exogenous testosterone. In contrast, GSI did not differ between C and I at 16 wk (P > 0.05), suggesting that, despite elevated anti-GnRH antibody concentrations, the antibodies induced may have been insufficient in titer or neutralizing capacity to fully suppress testicular development. This contrasts with the GSI reduction at the same dose in our preliminary experiments and, together with the variable testicular size in I (Fig. 1A), suggests considerable individual variation in the response. Quaresma et al. (2017) similarly reported that Improvac reduced serum testosterone without reducing testicular mass in broilers and suggested that the protocol and dosage could be improved.

Body weight was higher in C than in I and T from 7 wk onward (P < 0.01), and I was heavier than T between 11 and 15 wk (P < 0.05). Despite these weekly differences, overall ADG did not differ among groups (C: ∼20 g/d; I and T: ∼19 g/d; P > 0.05), and feed intake and FCR, which were measured per pen, were similar among groups, although T showed numerically lower feed intake and higher feed efficiency. Thus, unlike in swine, in which Improvac improves growth performance (Dunshea et al., 2001), immunocastration did not meaningfully affect overall productive performance, possibly reflecting differences in growth trajectory and energy partitioning between pigs and chickens.

Muscle color differed among groups only in the thigh: L* was lower in T than in I in both the ITL and the medial surface of the thigh (P < 0.05), and a* was higher in T than in I on the medial surface (P < 0.01), suggesting darker and redder coloration with testosterone supplementation. This may reflect effects of testosterone on myoglobin content or oxidative fiber proportion, which are higher in the thigh than in the predominantly glycolytic breast muscle. Moisture content did not differ among groups in either muscle, indicating that the differences in shear force described below are unlikely to be attributable to differences in muscle hydration.

Raw shear force of the ITL was lower in I than in both C and T (P < 0.05 and P < 0.001, respectively; Fig. 2A), whereas cooked shear force did not differ among groups. This suggests that immunocastration-associated testosterone deficiency may reduce connective tissue stiffness in thigh muscle, possibly through attenuation of collagen cross-linking or fibril organization (Purslow, 2005). The convergence of shear force values after cooking, which denatures collagen, is compatible with this interpretation, although collagen content and cross-linking were not measured directly. Shear force did not differ among groups in the breast muscle in either the raw or cooked state (P > 0.05), consistent with the generally lower connective tissue content of this muscle (Purslow, 2005).

**Fig. 2:**
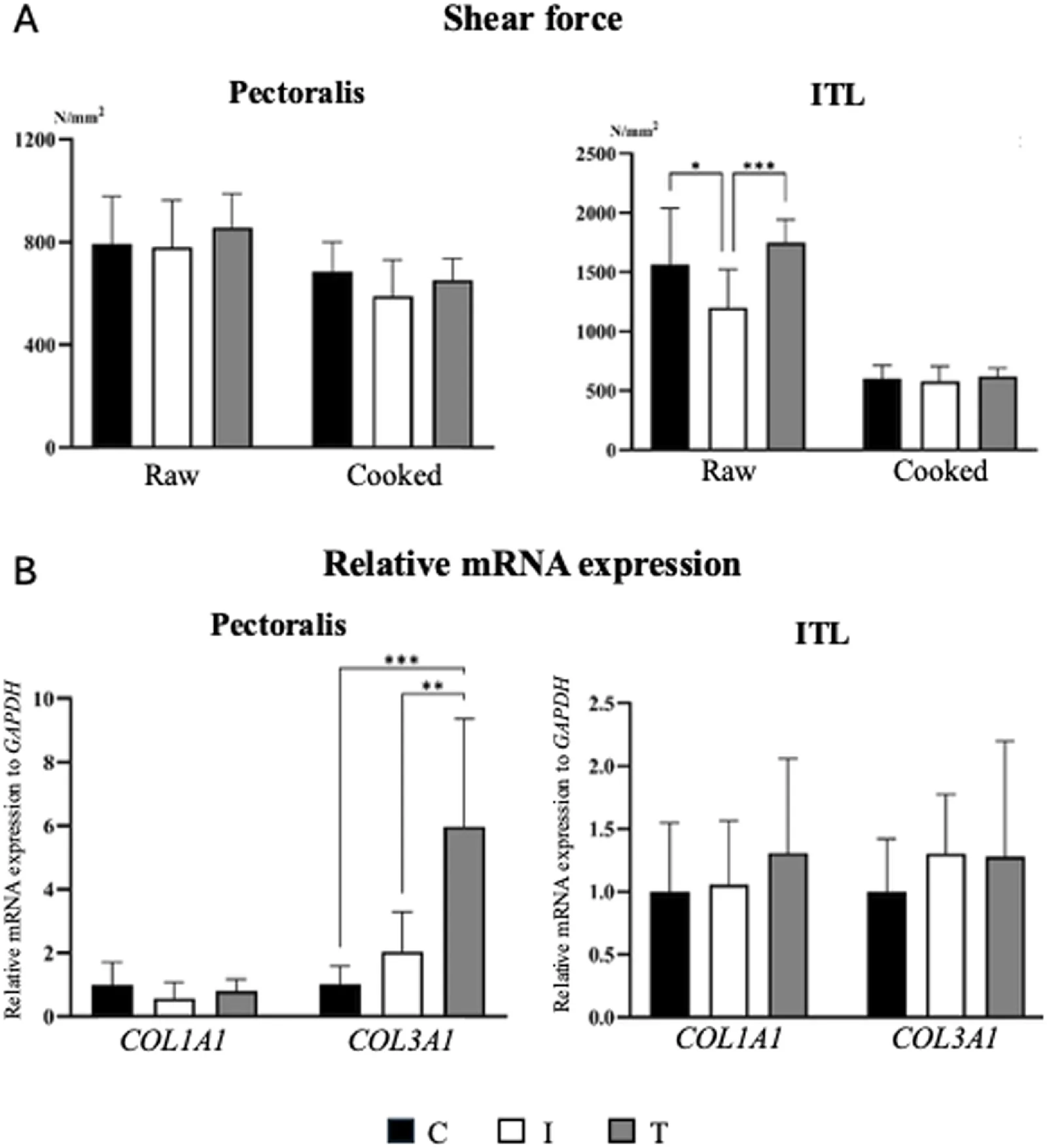
Shear force and collagen gene expression in breast (pectoralis) and thigh (ITL) muscles of male Sakura chickens at 16 wk of age. (A) Shear force (N/mm²) of the breast muscle (pectoralis) and the ITL measured before (raw) and after cooking (n = 7 per group). (B) *COL1A1* and *COL3A1* mRNA expression relative to *GAPDH* in the breast muscle and the ITL (n = 4 per group). Data are presented as mean ± SEM. Asterisks indicate significant differences among groups (Tukey’s test, *P < 0.05; **P < 0.01; ***P < 0.001). C, control; I, immunocastration; T, immunocastration plus testosterone supplementation.

In the breast muscle, *COL3A1* mRNA expression was elevated in T compared with C and I (P < 0.001 and P < 0.01, respectively; Fig. 2B), whereas *COL1A1* expression did not differ among groups (P > 0.05). No differences in *COL1A1* or *COL3A1* expression were detected in the ITL. Thus, shear force and collagen gene expression were dissociated in both muscles. This dissociation may reflect the distinct connective tissue biology of these muscles: in the ITL, a red muscle with high connective tissue content, reduced androgen signaling may have altered collagen cross-linking or fibril organization without changing steady-state collagen mRNA levels, whereas in the breast muscle, testosterone-driven *COL3A1* upregulation may reflect an early remodeling response that had not yet translated into measurable mechanical changes at 16 wk, because newly synthesized collagen requires time to mature and form stable cross-links. However, mRNA abundance does not necessarily reflect collagen content, post-translational modification, or cross-linking, which are the primary determinants of the mechanical properties of intramuscular connective tissue (Purslow, 2005). Because these parameters were not measured in the present study, the proposed link between collagen gene expression and shear force remains speculative and should be tested by direct measurements such as hydroxyproline content, collagen fiber organization, and cross-linking status.

Taken together, these findings indicate that Improvac induces a detectable anti-GnRH antibody response in male Sakura chickens and, particularly when combined with testosterone supplementation, is associated with suppressed testicular development, and suggest that exogenous testosterone may modulate collagen gene expression in a muscle-specific manner. The limited efficacy of the current protocol in suppressing testicular development in the I group underscores the need to optimize vaccination timing, dose, adjuvant formulation, and booster interval specifically for chickens, because the avian immune system differs from that of mammals in B-cell differentiation and antibody repertoire generation (Sayegh et al., 2000). From a practical standpoint, the present protocol, which requires rearing males for several weeks before vaccination, does not by itself overcome the economic disadvantage of rearing layer-type males, whose growth rate and feed efficiency are inferior to those of broilers (Murawska and Bochno, 2007). The present results should therefore be regarded as basic biological information rather than evidence of commercial feasibility; any practical application would require separate economic evaluation. The present study also has several limitations, including the absence of a testosterone-only group, the lack of a baseline hormone measurement before supplementation, and the small number of birds analyzed for hormones and gene expression; because these birds were selected on the basis of median GSI, within-group biological variation may have been underestimated, and these results should be interpreted with caution. Future studies incorporating a testosterone-only group, sample sizes determined by a priori power analysis, histological assessment of connective tissue architecture, quantitative collagen protein measurement including hydroxyproline content and cross-linking analysis, androgen receptor expression profiling, and consumer sensory evaluation will be necessary to fully characterize the impact of immunocastration on chicken meat quality.

## Declaration of AI and AI-assisted technologies in the writing process

During the preparation of this work, the authors used Claude (Anthropic) to assist with English language editing. After using this tool, the authors reviewed and edited the content as needed and take full responsibility for the content of the publication.

## Ethics approval

All experimental procedures were approved by the Animal Experiment Ethics Committee of Kyushu University (approval numbers: A24-348 and A25-376).

## Consent for publication

All authors approved the manuscript and its submission.

## Use of AI-assisted technologies

During the preparation of this work, the authors used Claude (Anthropic) to assist with English language editing. After using this tool, the authors reviewed and edited the content as needed and take full responsibility for the content of the publication.

## Author contributions

X. Zhang performed the animal experiments, measured growth performance, meat quality traits, serum hormone and antibody concentrations, and collagen gene expression, analyzed and interpreted the data, and drafted the manuscript. K. Hoshina and M. Uemori performed the animal experiments and collected samples, contributed to data acquisition, and critically reviewed the manuscript for important intellectual content. S. Egawa and S. Nishimura contributed to the experimental design and the interpretation of the results, provided resources for the study, and critically revised the manuscript for important intellectual content. Y. Z. Hosaka conceived and designed the study, supervised the experiments, interpreted the data, and critically revised the manuscript for important intellectual content. All authors read and approved the final version of the manuscript and agree to be accountable for all aspects of the work.

## Disclosures

The authors declare no conflict of interest.
